# A Decade-Long Review of the Virulence, Resistance, and Epidemiological Risks of *Klebsiella pneumoniae* in ICUs

**DOI:** 10.3390/microorganisms12122548

**Published:** 2024-12-11

**Authors:** Tao-An Chen, Ya-Ting Chuang, Chieh-Hui Lin

**Affiliations:** 1 Division of Respiratory Therapy, Department of Chest Medicine, Show Chwan Memorial Hospital, Changhua 500, Taiwan; b117100045@tmu.edu.tw; 2 Surgical Intensive Care Unit, Show Chwan Memorial Hospital, Changhua 500, Taiwan; s09901383@cjc.edu.tw; 3 Department of Chest Medicine, Show Chwan Memorial Hospital, Changhua 500, Taiwan

**Keywords:** *Klebsiella pneumoniae*, carbapenem-resistant organisms, intensive care units, multidrug resistance, virulence factors

## Abstract

*Klebsiella pneumoniae*, a major opportunistic pathogen, causes severe infections in both community and healthcare settings, especially in intensive care units (ICUs), where multidrug-resistant (MDR) strains, such as carbapenem-resistant *K. pneumoniae* (CRKP), pose significant treatment challenges. The rise in hypervirulent *K. pneumoniae* (hvKP) with enhanced virulence factors complicates management further. The ST11 clone, prevalent in China, exhibits both resistance and virulence traits, contributing to hospital outbreaks. ICU patients, particularly those with comorbidities or prior antibiotic exposure, are at higher risk. Treatment is complicated by limited antibiotic options and the increasing prevalence of polymicrobial infections, which involve resistant pathogens like *Pseudomonas aeruginosa* and *Acinetobacter baumannii*. Combination therapies offer some promise, but mortality rates remain high, and resistance to last-resort antibiotics is growing. Infection control measures and personalized treatment plans are critical, alongside the urgent need for vaccine development to combat the rising threat of *K. pneumoniae*, particularly in vulnerable populations. Effective management requires improved diagnostic tools, antimicrobial stewardship, and innovative treatment strategies to reduce the burden of this pathogen, especially in resource-limited settings. This review aims to provide a comprehensive analysis of the virulence, resistance, and epidemiological risks of *K. pneumoniae* in ICUs over the past decade, highlighting the ongoing challenges and the need for continued efforts to combat this growing threat.

## 1. Introduction

*Klebsiella pneumoniae* is an important opportunistic pathogen responsible for various infections in both community and healthcare settings [1,2,3]. It can cause severe infections affecting multiple organs, including the lungs, urinary tract, bloodstream, and wounds, thereby posing a significant threat to patient safety, particularly among vulnerable populations such as the elderly and immunocompromised individuals [1,2]. In recent years, the emergence of multidrug-resistant (MDR) strains of *K. pneumoniae* has escalated, presenting serious challenges to clinical management and treatment [4,5]. Carbapenem-resistant *K. pneumoniae* (CRKP) is a strain of *K. pneumoniae* that has developed resistance to carbapenem antibiotics, making infections difficult to treat. A study estimated that globally, at least 1.27 million people die annually from infections related to antimicrobial resistance (AMR), with *K. pneumoniae* being the second leading cause of mortality from antimicrobial pathogens. Consequently, the World Health Organization (WHO) has classified CRKP as a critical pathogen, emphasizing the need for strengthened infection control measures and precise treatment strategies [1,6].

The rise in CRKP in intensive care units (ICUs) is particularly concerning, with significantly high rates of infection and mortality [7,8]. Risk factors for CRKP infections include invasive procedures, respiratory support devices, prolonged hospital stays, and prior antibiotic use [2,3]. The challenges in treating CRKP are compounded by the limited availability of effective antibiotics, making it essential to identify these risk factors and implement stringent infection control measures [6,9].

Moreover, the adaptability of *K. pneumoniae* in various environments, including its ability to colonize human mucosal surfaces, facilitates its transmission and increases the likelihood of infections [10,11]. The WHO has prioritized the development of vaccines against *K. pneumoniae* as a key measure to combat the rising tide of antibiotic resistance and improve patient outcomes [1]. Understanding the epidemiology, virulence factors, and resistance mechanisms of *K. pneumoniae* is crucial for developing effective strategies to address this growing public health threat.

## 2. Virulence and Antimicrobial Resistance Mechanisms

### 2.1. Capsule

The capsule of *K. pneumoniae* is a critical virulence factor, closely associated with its hypermucoviscosity (HMV) and high virulence traits [12,13]. This capsule, composed of acidic polysaccharides, forms an outer protective layer that not only supports the hypermucoviscous phenotype but also confers resistance against host immune responses. Early studies demonstrated that capsule-deficient strains exhibit reduced pathogenicity in mouse models, showing lower bacterial densities in the lungs and bloodstream [14,15]. Capsule expression enhances the resistance of *K. pneumoniae* to immune defenses, enhancing survival and persistence within the host. It protects against phagocytosis, complement attacks, and antimicrobial peptides, primarily serving as a passive defense rather than actively inhibiting immune cells [12,16].

Geographically, capsule types K1 and K2 have distinct distributions, with K1 predominating in Asia and linked to sequence type (ST) 23, while K2 is associated with ST25, ST86, ST375, and ST380 [12,13]. Components like sialic acid in the capsule enhance both the hypermucoviscous phenotype and anti-phagocytic properties, bolstering immune evasion during infection [12]. The capsules are encoded by *wzi*, *wza*, *wzb*, *wzc*, *wzx*, and *wzy* [13,14]. A review study expanded on the regulatory mechanisms of the hypermucoviscous phenotype, noting that the overexpression of regulatory genes such as *rmpA*, *rmpA2*, *RcsB*, *KvrA* and *KvrB* boosts capsule synthesis, which is influenced by environmental factors. For example, under hypoxic conditions, the fumarate nitrate reduction regulator (FNR) reduces the expression of *rmpA* and *rmpA2*, thereby decreasing capsule polysaccharide synthesis [17]. Additionally, signals like glucose can stimulate capsule production and reduce cytokine secretion by peripheral blood mononuclear cells (PBMCs) in diabetic patients, which may further weaken immune clearance of the infection [18].

Although there is a strong association between the capsule and HMV, recent findings suggest that HMV is not entirely reliant on excessive capsule production. Genes like *RmpC* maintain the hypermucoviscous phenotype even when capsule synthesis is inhibited, indicating independent biosynthetic pathways for HMV and capsule synthesis [12]. Moreover, a study indicates that HMV is influenced by other factors, including iron uptake genes and *pagO* [13].

The emergence of hypervirulent *K. pneumoniae* (hvKP) strains, associated with HMV and a thick polysaccharide capsule, has introduced new challenges. HvKP strains, commonly linked to capsule types K1 and K2, exhibit enhanced virulence and can cause severe invasive infections even in healthy individuals, often leading to liver abscesses, meningitis, and endophthalmitis [12,13]. These hvKP strains contain regulatory genes such as *rmpA* and *rmpA2*, often found on virulence plasmids, which also harbor additional virulence factors, including iron acquisition systems like *iro* and *iuc*. K2-type capsules further aid hvKP in immune evasion by incorporating sialic acid residues, which mimic host cells [1]. Clinically, hvKP infections pose significant challenges due to both virulence and resistance. Although hvKP is typically antibiotic-sensitive, resistance, including carbapenem-resistant hvKP (CR-hvKP) strains, has been rising, complicating treatment options [12,13].

One promising treatment strategy is to use capsule depolymerases to degrade the capsule and reduce the immune resistance of hvKP. For example, phage-derived enzymes like RAD2 have shown potential in targeting and degrading the K2 capsule, enhancing immune clearance [19]. Such strategies that target unique hvKP virulence factors provide new directions for combating infections caused by hypervirulent, multidrug-resistant *K. pneumoniae* (MRKP) strains.

### 2.2. Siderophore Systems for Iron Acquisition

Iron is essential for bacterial metabolism, but its limited availability in the external environment presents a challenge for bacterial iron acquisition, serving as a nonspecific immune defense mechanism [12,13]. To survive and proliferate, bacteria such as *K. pneumoniae* synthesize and secrete small iron-binding molecules known as siderophores to secure this vital nutrient, as free Fe^3+^ is generally insoluble under physiological conditions. These iron–siderophore complexes are recognized by specific outer membrane receptors, facilitating transport into the periplasm and ultimately the cytoplasm through ABC transporters, where ferric iron is reduced to ferrous iron [12].

Research has shown that hvKP significantly enhances siderophore production compared to classical strains, exhibiting a six- to ten-fold increase, and expresses various siderophores including enterobactin, yersiniabactin, salmochelin, and aerobactin [12,13]. These virulence factors not only aid in bacterial survival in low-iron environments but also promote pathogenicity. Further studies indicate that the iron acquisition synthesis genes (such as *ent*) and accessory genes (like *ybt*, *iuc*, and *iro*) are highly expressed in hvKP, particularly the *iuc* and *iro* genes, which are rarely found in classical strains [20].

Additionally, a recently discovered protein known as *IroP*, encoded on the virulence plasmid of hvKP, suppresses the expression of type 3 fimbriae. *IroP* itself is regulated by iron levels, and research has shown that iron not only influences hypermucoid capsule production but also inversely regulates type 3 fimbria expression through *IroP* [21]. This evolutionary transcriptional switch may significantly contribute to the evolutionary success of hvKP, allowing it to adapt to varying nutrient environments while enhancing its virulence and ability to cause disease [21].

Recent research has analyzed the FepA homologs in *K. pneumoniae*, which play a critical role in the recognition and transport of iron–siderophore complexes [12,22]. In the *Kp52.145* strain, the identification of different receptors (such as IroN) responsible for recognizing and transporting ferric siderophores has provided new insights into how bacteria acquire iron [22]. These findings underscore the importance of iron acquisition systems in the virulence of *K. pneumoniae* and offer potential targets for novel antivirulence strategies.

### 2.3. Biofilms

*K. pneumoniae* is a significant pathogen known for its ability to form biofilms, which play a crucial role in its virulence and resistance to treatment. Biofilms are complex, structured communities of bacteria encased in an extracellular matrix that enhances resistance to antimicrobials and immune responses, such as the complement system and phagocytosis [23,24]. This structural feature contributes to the pathogenesis of various bacterial infections, with biofilm-related infections accounting for approximately 65–80% of all bacterial infections [23]. In *K. pneumoniae*, biofilm formation begins with adherence to surfaces, followed by microcolony development and maturation, which are facilitated by adhesins, fimbriae, and flagella [25]. These mature biofilms not only serve as reservoirs for pathogens but also exhibit polymicrobial interactions, particularly with species like *Pseudomonas aeruginosa*, which can further enhance resistance through interspecies dynamics [23,26].

Recent studies have elucidated the intricate relationship between biofilm formation, virulence factors, and antibiotic resistance in *K. pneumoniae* strains. Research indicates that strains isolated from clinical samples, particularly sputum, demonstrate varying biofilm-forming capabilities and resistance profiles. Notably, classical *K. pneumoniae* (cKP) strains show significantly higher levels of multidrug resistance compared to hvKP, which, despite being less resistant overall, possess virulence genes that enhance their pathogenic potential [24]. Specific genes related to iron acquisition, such as *entB* and *kfu*, are often associated with hvKP’s heightened virulence, facilitating survival and persistence in the host [25]. Moreover, the presence of extended-spectrum beta-lactamase (ESBL) genes correlates with both biofilm production and antibiotic resistance, particularly in cKP strains [23,27,28].

In summary, biofilms are a vital virulence factor for *K. pneumoniae*, providing both structural protection and contributing to antibiotic resistance. Understanding the dynamics of biofilm formation and its relationship with resistance genes is essential for developing effective treatment strategies against *K. pneumoniae* infections. Ongoing research continues to highlight the complexity of these interactions and their implications for clinical management [23,29].

### 2.4. Secretion Systems

Bacterial secretion systems play a crucial role in the survival and interaction of bacteria within their environments. Living in densely populated communities, bacteria must compete for resources such as nutrients and space. These secretion systems, located on the bacterial cell surface, allow bacteria to communicate with their surroundings, acquire essential nutrients, and transport effector proteins, thereby influencing interactions with other organisms, including hosts. In Gram-negative bacteria, nine distinct secretion systems have been identified, numbered from Type I (T1SS) to Type IX (T9SS), each serving unique functions [30]. For instance, Type VII (T7SS) and Type VIII (T8SS) are primarily involved in secreting pili and fibers, while Type IX (T9SS), a recently discovered system, is found exclusively in Bacteroides species [31].

*K. pneumoniae* has evolved several virulence mechanisms, including the Type VI Secretion System (T6SS). T6SS, discovered in 2006, is a key player in *K. pneumoniae*’s virulence arsenal, facilitating bacterial competition, host invasion, and immune evasion [12,30,32]. T6SS acts like a “nanoneedle”, injecting effector proteins such as Hcp and VgrG, which disrupt host cell physiology and enable *K. pneumoniae* to outcompete other microbes [12,13]. This system is particularly prominent in hvKP, which exhibit enhanced virulence and resistance mechanisms compared to cKP [12,33].

In recent years, extensive research has shown that T6SS contributes to *K. pneumoniae*’s ability to colonize and invade host tissues, especially in the gastrointestinal tract, and has been implicated in biofilm formation, which is crucial for persistent infections [12,13,30]. For example, a study on ST147, a rapidly spreading and extensively drug-resistant *K. pneumoniae* clone, highlighted T6SS’s role in competitive survival and colonization, as well as its association with virulence factors like fimbria expression, siderophores, and capsule formation [33]. Furthermore, T6SS enhances the pathogen’s resistance to antimicrobial agents, with some studies suggesting that antibiotic exposure may trigger T6SS activity, facilitating both the invasiveness and horizontal gene transfer of antibiotic resistance [12,30].

The molecular regulation of T6SS in *K. pneumoniae* is complex, with environmental factors like osmotic pressure and temperature influencing its expression [12,30]. Key regulatory proteins, including PhoQ and OmpR, modulate the activity of T6SS, ensuring its optimal function during infection and competition [12,30]. This regulatory flexibility allows *K. pneumoniae* to adapt to diverse environments, contributing to the success of hypervirulent strains in clinical settings. While much has been learned about the roles of T6SS in *K. pneumoniae* pathogenicity, antibiotic resistance, and interspecies competition, there remain unanswered questions about the precise mechanisms by which T6SS effectors are delivered and target specific host cells, suggesting the need for further research [30].

Given its central role in the pathogenicity and resistance of *K. pneumoniae*, T6SS represents a promising target for novel therapeutic strategies aimed at disrupting bacterial competition, reducing virulence, and controlling the spread of MDR strains [30]. However, more research is needed to fully understand the regulation and function of T6SS in *K. pneumoniae* and to identify effective ways to inhibit its activity, offering hope for combating infections caused by this increasingly problematic pathogen.

## 3. Genomic Study of *K. pneumoniae* in ICUs and CR-hvKP

The ST11 clone is particularly widespread in China and is known for acquiring virulence-associated genes, which may contribute to the emergence of hvKP, thereby enhancing MDR and pathogenicity [6]. A significant number of ST11 CRKP isolates in the study carried resistance genes, such as *blaKPC-2*, *blaTEM-1B*, and *blaCTX-M-65*, suggesting a convergence between virulence and resistance traits [6,34]. The primary mechanisms of resistance in carbapenem-resistant Enterobacteriaceae (CRE), including *K. pneumoniae*, are the production of carbapenemases, such as KPC, NDM, and OXA-48-like enzymes. These mechanisms are a major global concern as they often lead to high resistance against carbapenems, which are critical last-resort antibiotics used to treat severe infections [35,36]. In Europe, CRKP strains are especially prevalent in the Mediterranean and Balkan regions, with resistance rates up to 60% in Greece and 40% in Italy. These high resistance rates are largely due to the widespread presence of carbapenemases such as KPC, NDM, and OXA-48-like enzymes [37,38]. Whole-genome sequencing revealed that ST11 isolates shared a similar profile of virulence factors, including enterobactin, mucoid regulator (*rmpA*), and yersiniabactin, and exhibited resistance to multiple antibiotics, including carbapenems, cephalosporins, and aminoglycosides [6]. A study analysis of transmission dynamics revealed that, from 2021 onwards, CRKP isolates were primarily transmitted from the ICU to other hospital departments, with notable interdepartmental transmission events in 2023. The ICU served as the major reservoir for CRKP, which spread to other units, including oncology and neurology [39]. These studies emphasize the need for targeted infection control measures in high-risk areas like the ICU to mitigate the spread of MRKP strains, and advocate for further research to assess the full scope of transmission dynamics through environmental and healthcare worker samples.

The emergence of CR-hvKP poses a significant threat to public health, particularly in hospital settings where it is associated with severe infections such as pneumonia and intracranial infections. A study conducted in South China in 2023 found that KPC-2-producing CR-hvKP strains, especially those of ST11 and ST65, were dominant among hospital isolates. These strains harbor specific virulence factors, such as *rmpA*, *rmpA2*, *iucA*, *iroB*, and *peg-344*, with *rmpA* and *rmpA2* playing crucial roles in defining the hypervirulent nature of CR-hvKP [40,41,42]. The primary resistance mechanisms in these CR-hvKP strains are attributed to the production of carbapenemases, such as KPC-2 and NDM, with KPC-2 being the most common among *K. pneumoniae* isolates in China [35]. Tracheal intubation was identified as an independent risk factor for CR-hvKP infections, underscoring the importance of heightened vigilance, especially for elderly patients with underlying conditions [41,42]. Molecular epidemiological studies confirmed that the ST11 clone is the predominant genotype of CR-KP in China, indicating the widespread transmission of virulence genes within this clone, which poses a significant challenge for infection control measures [43].

Recent research has shown that the prevalence of *blaOXA-232*, especially in pediatric *K. pneumoniae* isolates, is increasing in China. This shift in the carbapenemase landscape suggests that OXA-232, previously rare, may become a more prominent resistance factor alongside KPC-2 and NDM in the future [35,44]. In Europe, OXA-48-like enzymes are prevalent in regions such as Turkey, Spain, France, Belgium, and parts of Africa and South America, but are relatively rare in the United States [45]. Despite the predictive power of virulence gene combinations, the study notes a lack of consensus on the optimal diagnostic criteria for CR-hvKP and calls for further research, including whole-genome sequencing, to improve diagnostic accuracy and better understand its virulence profile [41,46]. We summarize the genes associated with the aforementioned virulence and antimicrobial resistance mechanisms in Table 1.

## 4. Patient Vulnerabilities and Risk Factors for CRKP Infections in ICUs

### 4.1. Colonization and Prior Exposure to Antibiotics

A significant factor contributing to CRKP infections in ICU patients is prior colonization, particularly in patients admitted with existing microbial colonization of the gastrointestinal tract. Studies have consistently shown that ICU admission is an independent risk factor for the development of CRKP bloodstream infections (BSIs), with rectal colonization often detected upon admission [47,48]. Patients who are already colonized are at a significantly heightened risk of progression to infection, especially when additional risk factors, such as mechanical ventilation, central venous catheters, or prolonged hospital stays, are involved [48]. Colonization at multiple body sites or with other MDR organisms further complicates the situation, increasing the likelihood of polymicrobial infections and making treatment more challenging [49,50].

The use of broad-spectrum antibiotics, particularly carbapenems, is another critical factor in the development of CRKP infections. Carbapenems, while initially employed to manage infections caused by Gram-negative organisms, inadvertently promote the emergence and selection of carbapenem-resistant strains. In ICU settings, where antibiotics are frequently used, this creates a selective pressure that favors resistant pathogens, including CRKP [48]. The paradoxical relationship between prior carbapenem use and infection risk has been highlighted in various studies, indicating that overuse of carbapenems can initially suppress susceptible strains but lead to a rise in resistant pathogens [7,47]. This underscores the importance of antimicrobial stewardship and the need to minimize unnecessary antibiotic exposure in critically ill patients.

### 4.2. Comorbidities and Immune Suppression

ICU patients with underlying comorbid conditions, such as diabetes, cancer, liver disease, or immunosuppression, are particularly susceptible to CRKP infections [4,7,47]. Weakened immune responses in these patients hinder their ability to fight infections, allowing resistant organisms like CRKP to proliferate. A retrospective cohort study in Taiwan revealed that liver transplantation patients, particularly those with cirrhosis or hepatocellular carcinoma, are at an increased risk for CRKP infection [48]. The immunosuppressive therapy required to prevent organ rejection following liver transplants further exacerbates the risk of infection, contributing to poor clinical outcomes, including increased mortality.

In addition, the elderly population, often suffering from multiple chronic conditions, is also at increased risk for acquiring CRKP infections. A study of ICU admissions found that elderly patients were more likely to be colonized with CRKP and, consequently, develop subsequent infections [51,52,53]. This finding further emphasizes the need for heightened surveillance and infection control measures for older ICU patients.

## 5. Hospital Transmission Dynamics and Infection Control Challenges

### 5.1. Nosocomial Transmission and Outbreaks

In ICU settings, CRKP infections are primarily spread through nosocomial transmission [54,55,56]. Even with enhanced infection control measures, such as cohorting of infected patients, strict hygiene protocols, and environmental cleaning, outbreaks continue to occur, often with high mortality rates [52,55,57,58]. The ICU high-acuity environment, with its frequent use of invasive devices and high patient turnover, provides ample opportunity for pathogens to spread. A study has reported outbreaks in various countries, including Greece, where CRKP strains co-producing KPC-2 and VIM-1 carbapenemases were transmitted despite rigorous infection control efforts [58]. These outbreaks underscore the challenges of containing CRKP in hospital settings, where transmission can occur rapidly between patients, particularly in crowded or under-resourced ICUs.

One key mechanism behind the spread of CRKP in these environments is plasmid-mediated transmission, which allows the pathogen to rapidly acquire resistance to multiple antibiotics [51]. Studies have shown that CRKP strains, particularly those harboring carbapenemase-producing genes like *blaKPC*, are capable of persisting in the hospital environment and spreading across units [47,57]. Despite the implementation of infection control measures, such as hand hygiene, contact isolation, and environmental disinfection, the persistence of CRKP and other MDR organisms in the ICU setting remains a major concern.

### 5.2. Emergence of Hypervirulent Strains

The rise in hvKP strains in ICUs presents significant challenges in infection control and patient management. Studies from various regions highlight an increasing prevalence of hypervirulent strains, especially among patients with invasive procedures or prolonged antibiotic exposure [11,51,52]. For instance, the ST258 clone, which has shown high levels of resistance to multiple antibiotics, including third-generation cephalosporins and trimethoprim–sulfamethoxazole, was found to retain partial susceptibility to colistin, though with emerging resistance in 20% of isolates [11]. Such trends underscore the limited treatment options and the importance of rapid molecular detection for identifying resistant strains in critical care settings.

Colonization by *K. pneumoniae* in ICU patients is closely linked to subsequent infections, often with strains identical to the colonizers, indicating colonization as a key precursor to infection. In Vietnam, pathogenic strains like ST25, ST86, ST17, and ST23 have demonstrated increased virulence due to factors like siderophores and HMV genes [51]. Similarly, in China, patients colonized with CRKP at ICU admission were at a much higher risk of developing infections with strains carrying resistance genes such as *blaKPC-2*, stressing the importance of colonization screening to curb ICU-acquired infections [52].

Alarmingly, the ST11 CRKP strain, often associated with hypervirulent traits, has caused severe outbreaks in Chinese hospitals, with patients experiencing rapid mortality due to ventilator-associated pneumonia (VAP). This strain’s hypervirulence is linked to the acquisition of plasmids carrying virulence genes, increasing both survival in human immune cells and lethality in animal models [40]. Infection prevention and control interventions, including targeted bundle strategies and continuous monitoring, have shown promise in reducing CRKP incidence in ICUs, though sustained efforts remain necessary to prevent cross-transmission and mitigate the threats posed by these highly virulent and resistant strains [59].

In summary, ST11 is a dominant and highly virulent CRKP strain in China, while ST258 has become globally significant due to its widespread resistance patterns, particularly in European and Middle Eastern ICUs [11,40]. ST25, ST86, ST17, and ST23 are particularly notable in Vietnam, with specific virulence factors enhancing their pathogenicity [51]. To effectively manage the spread of these resistant and virulent strains in ICU settings, robust infection control measures, early screening for colonization, and ongoing molecular surveillance are essential. We present an outline of the regional distribution and specific characteristics of the strains listed in Table 2.

## 6. Therapeutic Challenges and Treatment Strategies for CRKP Infections

### 6.1. Limited Treatment Options and Combination Therapy

The treatment of CRKP infections in ICU patients is complicated by the limited efficacy of available antibiotics. Carbapenem-resistant strains of *K. pneumoniae* are often resistant to most classes of antibiotics, including beta-lactams, aminoglycosides, and even last-resort antibiotics like tigecycline and colistin [48,49]. For example, despite the tigecycline microbiological response rate of 70%, it has been associated with high ICU mortality rates, especially in patients with severe underlying comorbidities or superinfections [49]. The use of polymyxins like colistin remains a mainstay of therapy, but the increasing prevalence of colistin-resistant strains has rendered this option less reliable, leading to the need for alternative therapies [55].

Combination therapy has become the preferred treatment approach for CRKP infections in the ICU. Several studies have demonstrated the effectiveness of carbapenem-sparing combination regimens, such as the use of tigecycline, fosfomycin, and high-dose colistin for treating VAP caused by CRKP [48,49,60]. While combination therapies may offer improved outcomes compared to monotherapy, the success rate remains low, particularly in critically ill patients with coexisting infections and severe conditions, such as septic shock [61]. The high mortality associated with CRKP infections in the ICU emphasizes the need for more effective therapeutic options and the importance of personalized treatment plans based on microbiological data and the patient’s clinical condition [60].

### 6.2. Polymicrobial Infections and Resistance Mechanisms

The occurrence of polymicrobial infections, where CRKP is co-colonized with other MDR pathogens, further complicates treatment. ICU patients are particularly prone to polymicrobial infections due to their weakened immune systems and frequent use of invasive medical devices. In these cases, CRKP coexists with pathogens like *Pseudomonas aeruginosa*, *Acinetobacter baumannii*, or *Staphylococcus aureus*, making treatment even more challenging due to the diverse resistance mechanisms at play [62]. This polymicrobial nature of infections often necessitates broader-spectrum antibiotic coverage, which in turn increases the risk of further promoting resistance. Additionally, the use of ceftazidime–avibactam, a newer antibiotic option for treating CRKP, has been linked to the emergence of new resistance mechanisms, including metallo-beta-lactamase (MBL)-producing strains, further complicating the management of these infections [63].

## 7. Challenges of CRKP Infections in ICU

**Figure 1:**
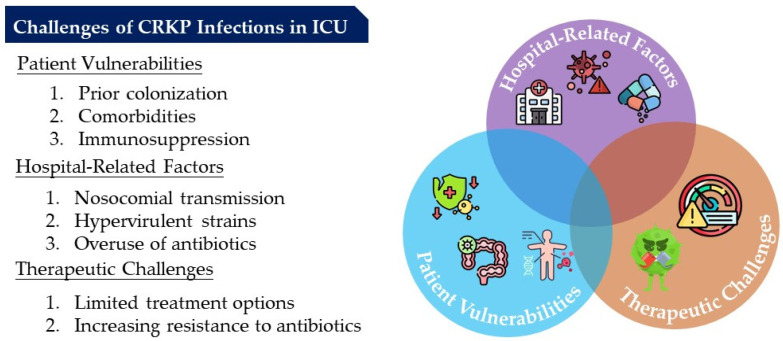
Challenges of CRKP infections in the ICU.

CRKP infections in the ICU are a multifaceted challenge, with several factors contributing to their emergence and poor outcomes [11,59]. Patient vulnerabilities, including prior colonization, comorbidities, and immunosuppression, play a central role in increasing the risk of infection [7]. Hospital-related factors, including nosocomial transmission, hypervirulent strains, and the overuse of broad-spectrum antibiotics, further exacerbate the problem. The therapeutic challenges of managing CRKP infections are compounded by limited treatment options, the rise in polymicrobial infections, and the increasing resistance to last-resort antibiotics [55,62,63]. A multifaceted approach, including rigorous infection control measures, antimicrobial stewardship, and the development of novel therapeutic options, is crucial to controlling the spread of CRKP in ICU settings. Continuous surveillance, tailored antibiotic therapies, and enhanced infection prevention strategies are essential to improving patient outcomes and mitigating the growing threat of MDR pathogens in the ICU. We present the challenges of CRKP infections in the ICU in Figure 1.

## 8. Urgent Need for Vaccine Development

*K. pneumoniae* is a major pathogen responsible for both community-acquired and healthcare-associated infections, posing a significant threat, particularly to neonates, infants, and adults with underlying conditions such as immunosuppression [7,64]. Globally, it is the second-leading pathogen linked to deaths attributed to AMR, exacerbating the public health challenge. Given the limited pipeline of new antibiotics and the rising issue of AMR, the development of vaccines against *K. pneumoniae* has become urgent. The WHO has prioritized the development of vaccines, particularly those targeting pregnant women, to protect newborns from *K. pneumoniae* infections [1]. A vaccine with 70% efficacy, if administered to pregnant women, could prevent nearly 400,000 cases of neonatal sepsis and 80,000 neonatal deaths annually [1,65]. Further research and economic evaluation are needed, especially in low- and middle-income countries (LMICs), to assess the vaccine’s impact on public health, healthcare burden, and societal costs [66]. Challenges in vaccine development include regulatory approval, funding, and equitable access, particularly in LMICs. Additionally, it is crucial to further analyze the pathways involved in the host response and their association with disease severity, as understanding these mechanisms is essential for the development of new therapeutic strategies and vaccines. In conclusion, the development of a *K. pneumoniae* vaccine is critical to reducing AMR-related deaths and diseases, requiring coordinated efforts from global health organizations, policymakers, and funding bodies, while also advancing our understanding of host–pathogen interactions to inform more effective vaccine strategies.

## 9. Conclusions

*K. pneumoniae* is a highly virulent and MDR pathogen, particularly problematic in ICU settings due to its ability to evade immune defenses and resist antibiotics through factors like its protective capsule, siderophore-mediated iron acquisition, biofilm formation, and plasmid-mediated resistance mechanisms. The rise in CR-hvKP, such as the ST11 clone, exacerbates the challenge, with these strains linked to severe infections and high mortality. Despite stringent infection control measures, CRKP transmission remains persistent in ICUs, facilitated by plasmid-driven resistance and the high turnover of critically ill patients. Colonization, prior antibiotic use, and comorbidities increase the risk of infection, highlighting the importance of early screening. Treatment options are limited, with combination therapies often necessary but not always effective, particularly in the presence of coexisting infections and emerging resistance. To address these challenges, more effective infection control, tailored therapies, and the urgent development of vaccines are crucial for curbing the spread of CRKP in hospital settings.

## Figures and Tables

**Table 1:** Genomic functions and virulence mechanisms of *K. pneumoniae*.

Virulence	Genes	Characteristics	References
Capsule	*wzi*, *wza*, *wzb*, *wzc*, *wzx*, *wzy*, *rmpA*, *rmpA2*, *rcsB*, *kvrA*, *kvrB*	Protective outer layer that evades immune response; enhances virulence and persistence in the host.	[1,13,14,17]
Siderophore Systems	*ent*, *ybt*, *iuc*, *iro*, *iroP*	Iron acquisition systems using siderophores for bacterial metabolism and survival in low-iron environments.	[20,21]
Biofilms	*entB*, *kfu*	Structured bacterial communities that provide resistance to antimicrobials and immune responses; play a key role in persistent infections.	[25]
Secretion System	*hcp*, *vgrG*, *phoQ*, *ompR*	Transport of effector proteins for bacterial survival, competition, and immune evasion, including the Type VI Secretion System (T6SS).	[12,13,30]
Carbapenem Resistance	*blaKPC-2*, *blaTEM-1B*, *blaCTX-M-65*, *blaOXA-232*	Resistance mechanisms against carbapenems, including carbapenemase production (e.g., KPC, NDM, OXA-48-like enzymes), affecting the treatment of severe infections.	[6,34,35,44]

**Table 2:** Geographical distribution and characteristics of hypervirulent *K. pneumoniae* strains.

**Region**	**Sequence Type**	**Characteristics**	**References**
Global (particularly in Europe and Middle East)	ST258	High resistance to antibiotics (e.g., third-generation cephalosporins), partial susceptibility to colistin.	[11]
China	ST11	Hypervirulent, linked to high mortality in VAP, carries virulence plasmids.	[40]
Vietnam	ST17	Increased virulence due to siderophores and HMV genes.	[51]
	ST23		
	ST25		
	ST86		

## Data Availability

The original contributions presented in the study are included in the article, further inquiries can be directed to the corresponding authors.
